# Orientation-dependent backbone-only residue pair scoring functions for fixed backbone protein design

**DOI:** 10.1186/1471-2105-11-192

**Published:** 2010-04-16

**Authors:** Andrew J Bordner

**Affiliations:** 1 Mayo Clinic, 13400 East Shea Boulevard, Scottsdale, AZ 85259, USA

## Abstract

**Background:**

Empirical scoring functions have proven useful in protein structure modeling. Most such scoring functions depend on protein side chain conformations. However, backbone-only scoring functions do not require computationally intensive structure optimization and so are well suited to protein design, which requires fast score evaluation. Furthermore, scoring functions that account for the distinctive relative position and orientation preferences of residue pairs are expected to be more accurate than those that depend only on the separation distance.

**Results:**

Residue pair scoring functions for fixed backbone protein design were derived using only backbone geometry. Unlike previous studies that used spherical harmonics to fit 2D angular distributions, Gaussian Mixture Models were used to fit the full 3D (position only) and 6D (position and orientation) distributions of residue pairs. The performance of the 1D (residue separation only), 3D, and 6D scoring functions were compared by their ability to identify correct threading solutions for a non-redundant benchmark set of protein backbone structures. The threading accuracy was found to steadily increase with increasing dimension, with the 6D scoring function achieving the highest accuracy. Furthermore, the 3D and 6D scoring functions were shown to outperform side chain-dependent empirical potentials from three other studies. Next, two computational methods that take advantage of the speed and pairwise form of these new backbone-only scoring functions were investigated. The first is a procedure that exploits available sequence data by averaging scores over threading solutions for homologs. This was evaluated by applying it to the challenging problem of identifying interacting transmembrane alpha-helices and found to further improve prediction accuracy. The second is a protein design method for determining the optimal sequence for a backbone structure by applying Belief Propagation optimization using the 6D scoring functions. The sensitivity of this method to backbone structure perturbations was compared with that of fixed-backbone all-atom modeling by determining the similarities between optimal sequences for two different backbone structures within the same protein family. The results showed that the design method using 6D scoring functions was more robust to small variations in backbone structure than the all-atom design method.

**Conclusions:**

Backbone-only residue pair scoring functions that account for all six relative degrees of freedom are the most accurate and including the scores of homologs further improves the accuracy in threading applications. The 6D scoring function outperformed several side chain-dependent potentials while avoiding time-consuming and error prone side chain structure prediction. These scoring functions are particularly useful as an initial filter in protein design problems before applying all-atom modeling.

## Background

Empirical scoring functions, or knowledge-based potentials, have been successfully applied to a wide variety of problems in biomolecular modeling. These scoring functions are derived from the statistics of residue or atomic interactions as observed in experimental protein structures. Most such scoring functions depend on amino acid side chain conformations, either through the definition of residue contacts [1-3] or through the dependence of the potentials on the distances between side chain atoms or residue centroids [4-6].

Another class of scoring functions, which includes the ones considered in this study, depend only on the protein backbone conformation. These scoring functions have two significant advantages: (1) they can be directly applied to proteins structures with unknown or uncertain side chain conformations and (2) they can be rapidly calculated. Because they are independent of the side chain conformations, these scoring functions can be applied to proteins for which the correct side chain conformations are not available from experimental structures. In such cases, computationally expensive and potentially inaccurate side chain optimization would be necessary in order to evaluate a side chain-dependent scoring function for the protein structure. Comparative modeling results have shown that although successful methods usually agree on the core backbone structure, they have considerably more difficulty in predicting correct side chain conformations [7]. Also, because backbone-only scoring functions do not require side chain optimization they can also be used to evaluate protein structures more quickly. Protein design applications require such a fast but approximate evaluation of protein stability for a large number of sequences. Because of the high-dimensional search space for side chain geometry optimization, empirical scoring functions that depend only on backbone geometry offer a computationally efficient solution to these tasks. Finally, even modeling problems in which a complete atomic structure of a protein is required can be efficiently accomplished using a multi-scale approach in which potential structures are first filtered using the fast backbone-only scoring function and the remaining structures then subjected to more time-consuming all-atom refinement and rescoring.

The non-uniform angular distributions of side chains about residues of different types observed in protein structures [8] suggests that residue pair scores that depend on the relative position and orientation of the residues will discriminate native from non-native protein structures better than scores that depend only on the residue separation distance. Some previously developed scoring functions incorporate this orientation dependence through the orientation of residue side chains [9-11]. The scoring functions derived in this study also incorporate orientation dependence but, unlike those scoring functions, they depend only on the protein backbone conformation. Two previous studies have examined orientation-dependent empirical potentials for backbone structures. The study of Onizuka *et al*. [12] defined local coordinate systems centered on C_*α *_atoms and fit residue pair potentials that were either functions of their relative position (3D) or their relative position and orientation (6D). Potentials were fit to data for different sequence separations using a truncated expansion in terms of spherical harmonics. The low-order terms included in the expansion yield a smoothed potential function that presumably eliminates sampling noise. A comparison between 1D (separation distance dependent), 3D, and 6D potentials with different expansion cutoffs showed that the 3D potential performed the best in ranking the native structure from among decoys. A later study by Miyazawa and Jernigan [13] also used similar spherical harmonic expansions in order to derive orientation-dependent potentials that were a function of backbone coordinates. Unlike Onizuka *et al*., these were residue contact potentials and so did not have any explicit dependence on the separation distance. More importantly, the residue contact potentials in Miyazawa and Jernigan are not strictly backbone-only potentials since they implicitly depend on side chain conformations via a smooth contact cutoff that is a function of the distances between the geometrical centers of the two residue side chains. Also, unlike Onizuka *et al*., Miyazawa and Jernigan concluded that accounting for the relative orientation of residue pairs through Euler angles using a 5D residue contact potential improved fold recognition performance over a 2D potential that accounts only for the relative positions of the contacting residue pairs. Whereas the scoring functions in those studies were tested using folding decoys, the scoring functions considered here are designed and tested for the different modeling problem of fixed backbone protein design. The appropriate decoy structures in this case are incorrect fixed backbone threading solutions with the correct backbone structure but incorrect amino acid sequences. The scoring functions described here are optimized to detect the correct (native) sequence from among many incorrect (decoy) sequences for a common backbone structure.

We have developed position and orientation-dependent residue pair scoring functions that depend only on the protein backbone structure, *i.e*. are completely independent of side chain conformations. As discussed above, these scoring functions are expected to be useful for protein design as well as fold recognition. 6D scoring functions that depend on both the relative position and orientation of the residue pairs were compared with 3D scoring functions, which depend only on the relative position, and with 1D scoring functions, which depend only on the separation distance. Also, the challenging task of estimating the high-dimensional residue pair distributions was solved using a different method, Gaussian Mixture Models (GMMs), than the spherical harmonic expansions employed in the two previous studies. One advantage of GMMs is that they are able to directly estimate the 3D and 6D residue pair distributions, including off-diagonal contributions from multiple orientational variables. Another advantage of the GMM formulation used in this study is that the optimal model complexity, or number of parameters, is estimated from the data using the Bayes Information Criterion (BIC) statistic [14].

The relative effectiveness of the 1D, 3D, and 6D scoring functions was evaluated by their ability to identify the correct threading solution from among a set of decoy threading solutions generated either by shuffling the native sequences or by cross-threading for a large non-redundant set of proteins. The new scoring functions were also compared with side chain-dependent empirical potentials from other studies using the same decoy set. Also, in order to illustrate the utility of the new backbone-only scoring functions two computational methods that exploit their advantages over traditional side chain-dependent potentials were studied. The first method involves averaging the residue pair scores over threading solutions for homologs. Because homologous proteins with significant sequence similarity usually adopt the same backbone structure such a procedure is expected to improve accuracy by combining scores for these distinct but structurally similar proteins. This method was applied to the difficult problem of predicting interactions between alpha helices in membrane proteins using fixed backbone threading and found to further improve accuracy. The second application of the new scoring functions involves using a fast approximate optimization method, called Belief Propagation, to estimate the optimal sequence for a given protein backbone structure. This design method was compared with one using all-atom modeling on a fixed protein backbone structure and found to be more robust to inevitable small backbone variations between the template and native protein structures.

## Results and Discussion

### Overview of the backbone-only residue pair scoring functions

The new backbone-only log-odds residue pair scoring functions were derived as the logarithm of the ratio between the backbone orientational distribution of residue pairs in correct native structures and the corresponding distribution for incorrect decoy structures, which corresponds to randomly shuffled amino acid sequences for the same native backbone structures. The latter background distribution is the same as the distribution of all residue pairs in protein structures, regardless of their type. Such a background decoy distribution is appropriate for the desired modeling application of fixed backbone protein design and threading but is non-optimal for other applications, such as *ab initio *protein folding. Also only residue pairs that are at least 6 residues apart in the protein sequence and with C_*β *_separations ≤ 10 Å were included in the scores. This eliminated residue pairs that were too close in the linear sequence so that their relative position and orientation are largely determined by the local secondary structure as well as residue pairs that were too far apart and so have little orientational preference because they only interact weakly. The 1D, 3D, and 6D scoring functions were derived based on the distributions of separation distances, relative positions, and both relative positions and orientations of particular residue type pairs, respectively. While the 1D scoring functions were fit using kernel density estimation (KDE), the difficult high-dimensional density estimation for the 3D and 6D scoring functions could not be accomplished by KDE so that another technique, Gaussian Mixture Models (GMMs), was employed.

### Prediction accuracy for individual residue type pairs

The performance of the scoring function was first evaluated for each of the 210 residue type pairs individually in order to find out which individual residue pairs make the largest contributions to the overall protein threading accuracy. The performance of each residue type score was assessed by the area under the Receiver Operating Characteristic (ROC) curve (AUC) for 10-fold cross-validation results. The ROC curve displays the tradeoff between sensitivity and specificity as the score cutoff is varied. Higher AUC values, near 1.0, indicate better prediction performance.

The cross-validation sets were created by randomly dividing the residue pair data for the non-redundant set of proteins into 10 approximately equal-sized sets such that all data for a particular protein is contained within only a single set. The predictions were then made on each set using log-odds scoring functions fit to data in the other 9 sets. This procedure then gives an estimate of the performance on novel data since the predictions are made for residue pair data from proteins unrelated to those used to fit the scoring functions.

The residue pairs whose 6D scores have the highest accuracy are Cys-Cys and all paired combinations of Iso, Leu, and Val. The high accuracy of the Cys-Cys score is due to the fact that a large percentage of such pairs (44%) form conformationally constraining disulfide bonds thus making their distinctive relative orientation easier to predict. Iso, Leu, and Val are some of the most commonly occurring residues, particularly in the protein core where they can form more contacts than residues on the surface. In fact, there is a significant correlation (Spearman's rank correlation coefficient *ρ *= 0.60, significance < 2.2 × 10^-16^) between the prediction accuracy, as measured by the AUC, and the number of training examples for each residue type pair. This means that generally the accuracy is better for common residue pairs than for rare ones, presumably because a large quantity of independent training examples are necessary in order to fit the high-dimensional probability densities. The median AUC over all 210 residue pairs was 0.60.

**Figure 1:**
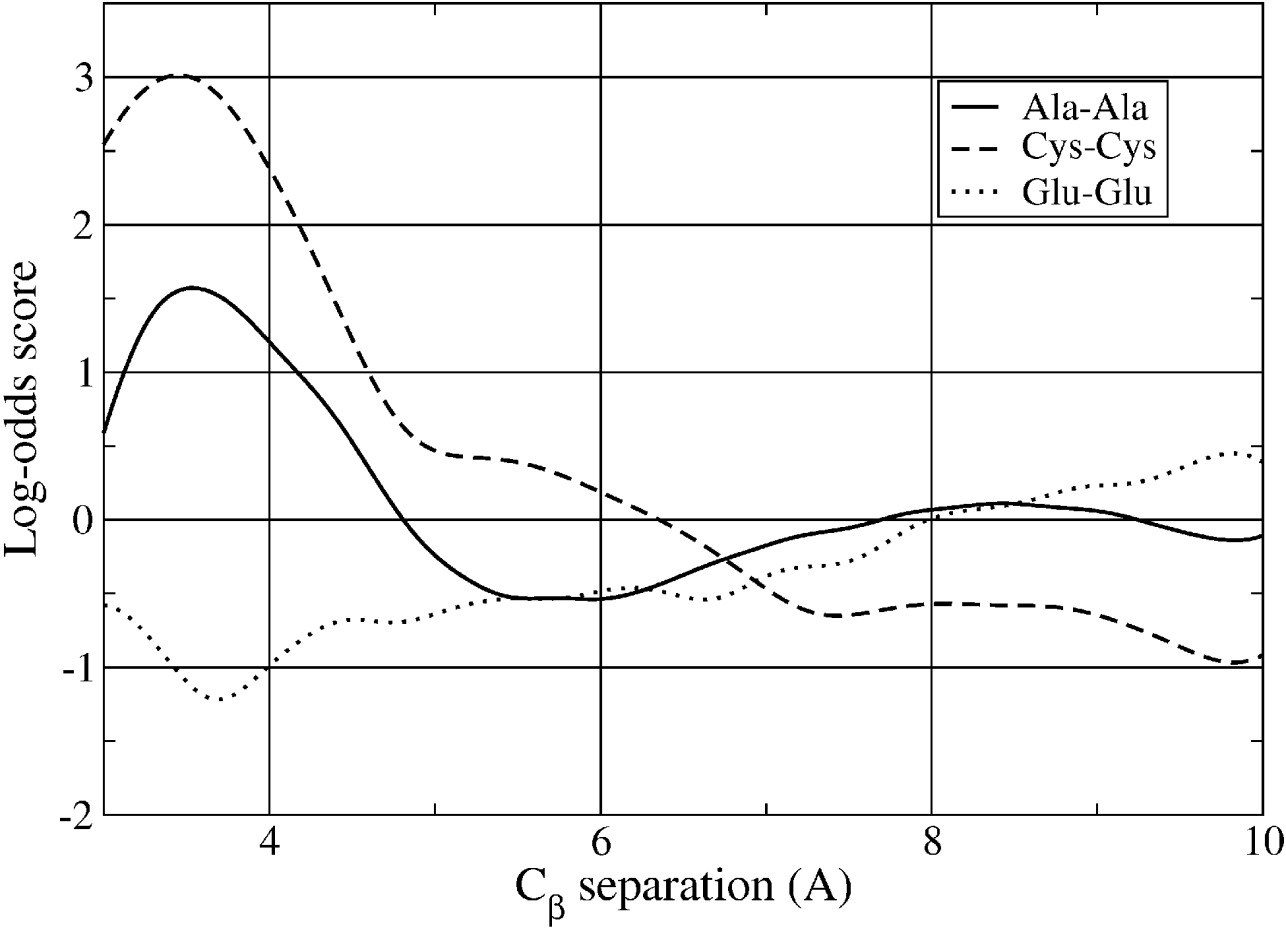
**1D log-odds scores as a function of C_*β *_separation for Ala-Ala, Cys-Cys, and Glu-Glu residue pairs**. The Cys-Cys function has a peak near the typical C_*β *_separation for disulfide bonds, in the range of 3.5-4.0 Å and is negative for large separations. On the contrary, the score for the same-charge Glu-Glu pairs is negative for small separations and positive for large separations, reflecting the electrostatic energy penalty for close proximity. Both the Cys-Cys and Glu-Glu scores are among the most accurate because of these physical constraints on their separations. The Ala-Ala score, shown for comparison, manifests an oscillatory behavior with a peak near that of the Cys-Cys score.

For comparison, the same analysis was also performed for the distance-dependent 1D scores. The results showed that some of the same residue pairs had the highest accuracy as for the 6D score, namely Cys-Cys as well as all combinations of Iso, Leu, and Val. However, in addition the same-charge residue pairs Glu-Glu and Asp-Glu were among the most accurately predicted residue pairs. This is likely due to the unfavorable electrostatic energy for close separations, which is reflected in negative scores at close separations for these residue pairs. Figure 1 shows a plot of the 1D residue pair scores for Cys-Cys and Glu-Glu and illustrates these trends as a function of inter-residue separation. The median AUC for the 1D scores was 0.53, which is lower than that for the 6D scores. As will be seen in the next section, the higher accuracy of the individual 6D residue scores translates into higher accuracy for total protein threading scores.

It is somewhat surprising that the accuracy of the individual residue pair scores was significantly correlated with the amount of training data even for the 1D scores (*ρ *= 0.49, significance = 5 × 10^-14^). This implies that there is still room for improvement in both the 1D and 6D scores and that their accuracy will likely increase as new experimental structures of proteins become available and provide additional independent training data.

### Threading prediction accuracy on a benchmark set

The accuracies of the residue pair scores were assessed in two ways: (1) by their ability to identify the native sequence of a protein from among a set of 5000 distinct decoy sequences and (2) cross-threading of all sequences in the benchmark set against a subset of the structures. In order to reliably estimate the prediction accuracy for novel data and to account for potential overfitting, 10-fold cross-validation was used for both test methods, thus insuring that the scoring functions were fit to a different set of protein structures than the set of proteins used to assess their accuracy. In the first test method, 4999 decoy sequences were generated for each protein in the benchmark set by shuffling the native sequence. Cross-validation predictions were then made for all 5000 sequences (native + decoy) and the rank of the native sequence determined. The results for this test are shown in Table 1. In the second test method, all-against-all gapless cross-threading was performed for the 333 structures in the first cross validation set using residue pair scoring functions fit to the remaining 2995 structures in the benchmark set. Only sequences with at least as many residues as the template structure were threaded and all possible gapless alignments were considered. This resulted in a total of over 52 million threading solutions for all protein structures. The number of possible threading solutions varied dramatically between structures and ranged from 43, for the largest template structure, to 1472929 for the smallest template structure. Because of this large variation, the fractional rank of the native sequence, or ratio of the rank of the native sequence to the number of threading solutions for the particular template structure, was reported, rather than the absolute rank of the correct (native) sequence. These results are shown in Table 2.

**Table 1:** A comparison of the accuracy of the new scoring functions with other residue pair scoring functions from the literature in detecting correct threading solutions

Residue pair scoring function	Rank 1 (0.02%)	Rank ≤ 50 (1%)	Rank ≤ 100 (5%)
**1D**	2357 (70.8%)	2945 (88.5%)	3030 (91.0%)
**3D**	2774 (83.4%)	3128 (94.0%)	3183 (95.6%)
**6D**	2911 (87.5%)	3200 (96.2%)	3231 (97.1%)

**MJ1999**	2714 (81.6%)	3033 (91.1%)	3068 (92.2%)
**TE2000**	2054 (61.7%)	2737 (82.2%)	2847 (85.5%)
**RMF2006**	623 (18.7%)	1632 (49.0%)	1906 (57.3%)
**RMF2008/6-bin**	1386 (41.6%)	2378 (71.5%)	2559 (76.9%)
**RMF2008/7-bin**	1396 (41.9%)	2391 (71.8%)	2548 (76.6%)

The 5000 threading solutions for each of the 3328 template structures were ranked using either the 1D, 3D, or 6D residue pair scores or five different empirical potentials with available published parameters. The empirical potentials evaluated were from Miyazawa and Jernigan 1999 [16] (**MJ1999**), Tobi and Elber 2000 (**TE2000**), Rajgaria, McAllister, and Floudas 2006 [15] (**RMF2006**), and Rajgaria, McAllister, and Floudas 2008 [17] (**RMF2008**). The values indicate the number of protein structures for which the rank of the native sequence was within the indicated percentile. The corresponding percentage of the total structures is given in parentheses.

**Table 2:** Prediction results for all-against-all gapless cross-threading of 333 proteins

Residue pair scoring function	Percentile rank of native sequence			Median percentile rank
		
	0.001%	0.01%	0.1%	
**1D**	118 (35.4%)	209 (62.8%)	261 (78.4%)	2.47 × 10^-3^
**3D**	121 (36.3%)	240 (72.1%)	286 (85.9%)	1.98 × 10^-3^
**6D**	125 (37.5%)	247 (74.2%)	293 (88.0%)	1.66 × 10^-3^

The number of structures for which the percentile rank of the native sequence was less than or equal to the indicated cutoffs are given in columns 2-4 and the median percentile rank is given in the last column. The corresponding percentage of the total structures is given in parentheses. The threading solutions were ranked using the indicated residue pair scoring function.

### Score accuracy improves with increasing dimension

Both the results for shuffled sequences, in Table 1, and the results for cross-threading, in Table 2, show that the prediction accuracy steadily improves as the dimension of the scoring function increases from 1D to 3D to 6D. Although the largest incremental improvement occurred in going from 1D to 3D potentials, the highest dimensional 6D potentials still had significantly higher accuracy than the 3D potentials in both tests. This conclusion contrasts with that of Onizuka *et al*. [12], who found that their 3D potentials achieved higher accuracy than their 6D potentials. However, this result agrees with the general conclusion of Miyazawa and Jernigan [13], although as mentioned above, the contact potentials in that study differ from the backbone-only scoring functions considered here.

### Comparison with other empirical potentials

The accuracy of the backbone-only scoring functions was also compared with that of one other backbone-only potential, that of Rajgaria, McAllister, and Floudas 2006 [15] (RMF2006), as well as with several conventional side chain-dependent empirical potentials. This was accomplished by calculating the total energies for the same threading benchmark set used to evaluate the backbone-only scoring functions. The results, as evaluated by the ranks of the native sequences, are given in Table 1. Side chain-dependent empirical potentials from the following studies were evaluated: Miyazawa and Jernigan 1999 [16] (MJ1999), Tobi and Elber 2000 (TE2000), and Rajgaria, McAllister, and Floudas 2008 [17] (RMF2008). These particular potentials were chosen because they all have available published parameters. The MJ1999 potential was the only residue contact potential while the other side chain-dependent potentials were a function of the side chain centroid separation distances. Side chain conformations were generated for the template backbone structures using the ROSETTA 3 program [18].

The results in Table 1 show that the MJ1999 contact potential was the most accurate among the five other empirical potentials. They also indicate that this potential achieves better performance than our 1D scoring function but worse performance than our 3D and 6D scoring functions. The other four empirical potentials all had lower accuracy on the benchmark set than even the 1D scoring function. This may be due to the fact that these four potentials were originally designed for different protein modeling tasks than fixed backbone design. The TE2000 potential was optimized to discriminate correct structures from decoys generated from a combination of gapless threading and all-atom structure prediction while the other three potentials (RMF2006, RMF2008/6-bin, and RMF2008/7-bin) were optimized to discriminate correct structures from among decoys generated from all-atom structure prediction. The background distribution for decoys generated by all-atom structure prediction is expected to be different from that for decoys generated by fixed-backbone threading, which is most relevant for this test. However, these results demonstrate that the 3D and 6D backbone-only scoring functions achieve accuracy that is competitive with side chain-dependent empirical potentials without requiring time-consuming prediction of side chain conformations prior to score evaluation.

### Examination of the difficult threading cases

The protein structures for which the most sensitive scoring function still gave poor threading results were examined in detail in order to determine common factors that may contribute to the difficulty of recognizing the correct solutions using the 6D score. For this purpose, all structures for which the 6D score assigned the native structure a rank greater than 1000 (20% percentile rank) were examined. A total of 22 structures satisfied this criterion. Five properties were overrepresented in these structures as compared with the other structures in the benchmark set. These distinctive characteristics of the structures were (1) solved by experimental methods other than X-ray diffraction (mostly solution NMR), (2) small protein domains, (3) large disordered regions, (4) subunit in a complex, and (5) few residue-residue contacts. These properties are not independent since NMR can only be used to solve relatively small proteins and also can be used to obtain structures of proteins with disordered regions, which by definition have few residue-residue contacts. A total of 18 of the structures were solved by NMR and one was a structure fit to electron microscopy data. This proportion of non-X-ray structures was significantly higher (p = 5.3 × 10^-8^, Fisher exact test) than in the set of other benchmark set structures, which included 973/3306 non-X-ray structures. A total of 15 protein structures contained significant disordered regions, which also had multiple conformations in the NMR structure ensembles. In addition, five of the proteins were subunits in a structure of a protein complex and so may be unstable as monomers. Finally, because many structures had little regular secondary structure (*α*-helix or *β*-strand), they also had relatively few residue-residue contacts compared to more structured, compact proteins. These properties are interdependent so that it is unclear whether the poor threading performance is because NMR structures may contain significant errors [19], or because they have few non-local residue-residue contacts that contribute to the total score, or because the disordered regions cannot be reliably recognized by the score.

### Combining the residue-pair scores of homologs improves threading accuracy

Proteins with significant sequence similarity usually have similar backbone structures [20-23]. This suggests an approach to improving threading accuracy by combining a potential threading solution with the corresponding solutions for homologous proteins. This can be accomplished by calculating the average over the score for the original threading solution and the scores for the homolog threading solutions, each obtained by replacing the native sequence segment with the aligned segment of a homolog sequence. This method is well suited to the backbone-only scoring functions because the scores can be quickly evaluated for the multiple homolog threading solutions. It should be noted that this procedure is applicable only to protein threading applications and not to protein design because it requires protein sequences that are evolutionarily related to the query sequence.

This procedure was tested using all-against-all cross-threading of a non-redundant set of contacting pairs of transmembrane *α*-helices in membrane proteins. This is a particularly challenging task for threading because of the limited number of inter-helical residue contacts, compared with the number of residue contacts in a typical complete protein structure. Cross-threading was performed for a non-redundant set of 78 contacting TM helices, which significantly differ both in sequence and structure. The effects of three different factors on the threading accuracy were investigated by calculating the percentile rank of the correct solution among all cross-threading solutions for each TM helix pair. First, the scores calculated by averaging the threading scores over homologs were compared with the usual scores calculated for just the native sequences. Second, the scores were calculated both with and without a membrane depth-dependent scoring function that reflects the propensity of each residue type to occur at a particular depth in the membrane. Third, the effect of the size of the TM helix pair interface was studied by calculating prediction statistics for only the 27 TM helix pairs that have at least 15 inter-helix residue contacts (defined by atomic separation < 4 Å). The results for all 78 TM helix pairs are shown in Table 3 and the results for the large interface helix pairs are in Table 4.

**Table 3:** Comparison of cross-threading results for 78 contacting transmembrane helix pairs using the 6D potential and optionally including homolog sequences or a membrane depth-dependent residue potential

Include homolog sequences?	Include depth-dependent score	Percentile rank of native sequence			Median Percentile Rank
			
		1%	5%	10%	
No	No	15 (19%)	29 (37%)	41 (53%)	8.4%
No	Yes	16 (21%)	31 (40%)	43 (55%)	8.0%
Yes	No	22 (28%)	38 (49%)	49 (63%)	6.0%
Yes	Yes	22 (28%)	39 (50%)	56 (72%)	5.1%

The backbone structure of each helix pair was used as a template and the amino acid sequences of all helix pairs were threaded into the structure in both possible helix correspondences, (A→ A', B→ B') and (A→ B', B→ A'), and in all possible ungapped alignments. The threading solutions for each template structure were ranked using the appropriate score and the percentile rank of the correct native sequence calculated. These results show that both including the homolog sequences and including the depth-dependent score improve the threading accuracy.

**Table 4:** Results for the same quantities shown in Table 3, except only for the 27 transmembrane helix pair structures that have at least 15 inter-helix residue contacts

Include homolog sequences?	Include depth-dependent score	Percentile rank of native sequence			Median Percentile Rank
			
		1%	5%	10%	
No	No	9 (33%)	13 (48%)	20 (74%)	6.7%
No	Yes	9 (33%)	15 (56%)	20 (74%)	3.6%
Yes	No	10 (37%)	13 (48%)	20 (74%)	5.3%
Yes	Yes	11 (41%)	16 (59%)	22 (81%)	2.3%

Comparison with the results in **Table 3 **show that the threading accuracy is higher for this subset of helix pairs with large interfaces, because of the stronger signal resulting from more inter-helix residue contacts.

The results in Tables 3 and 4 show that each of the three factors have clear effects on the threading accuracy. Most importantly, combining threading scores for homologs significantly improves the threading accuracy, as can be seen by the lower median percentile rank of the correct solution when homologs are included. As expected, including the depth-dependent scoring function also improves the accuracy because it provides information on the suitability of the threading solution that is independent of the residue pair score. Finally, the accuracy is considerably higher for the subset of helix pairs with more residue contacts. This can be explained by the fact that additional residue contacts provide more constraints on potential threading solutions and thus lead to better discrimination of correct solutions. A more extreme example of this trend can be seen by comparing the cross-threading results for the TM helix pairs with those for complete proteins shown in Table 2. The much lower percentile rank of the correct threading solutions, indicating higher accuracy, for complete proteins is likely due in large part to the much greater number of residue contacts in the larger complete proteins.

### Calculating the optimal sequence using Belief Propagation

Next, we demonstrate how the new backbone scoring functions can be applied to solving problems in protein design without computationally costly conformational sampling. One question that arises in the context of protein design and the study of protein folding is what amino acid sequences yield the most stable proteins with a given backbone structure. Based on the Boltzmann hypothesis of empirical threading potentials, which implies that the most commonly observed interactions in protein structures also have the lowest energy, this problem can be translated into finding amino acid sequences that maximize the total score for the backbone structure of interest. In this study we consider the contributions of single residues and residue pair interactions but not any higher order (≥ 3) residue interactions. The total score, *S*_total_, is then(1)

in which T_i _is the type of residue *i*, **P**_i _are its properties (here the solvent accessibility and secondary structure type), and **Ω**_ij _are the relative position and orientation between residues *i *and *j*. The same amino acid sequence that maximizes *S*_total _for a fixed backbone also maximizes its exponential(2)

Because the objective function, , is to be optimized with respect to the amino acid sequence  for a fixed backbone, its implicit dependence on the backbone geometry, through the **P**_i _and **Ω**_*ij *_parameters, is not shown.

The function in Eq. (2) has the same functional form as a pairwise Markov Random Field (MRF) model, up to a multiplicative constant 1/Z, where Z is the partition function. The partition function is independent of the amino acid sequence. Since only the sequence that maximizes Eq. (2) is of interest, and not its maximum value, this constant may be ignored.

The main advantage of interpreting the objective function in Eq. (2) as a MRF is that an efficient method, called Belief Propagation (BP) [24,25], can be used to solve it. There are two formulations of BP, the standard algorithm to calculate marginal distributions for a MRF and the max-product algorithm to calculate the maximum *a posteriori *(MAP) solution. Because we would like to find the sequence that maximizes Eq. (2), the latter max-product algorithm will be used. Eq. (2) can also be solved as an Integer Linear Programming problem [26], however BP offers a simple and fast alternative that generally yields accurate approximate solutions [27]. The MAP problem was solved by first converting the pairwise MRF corresponding to Eq. (2) into an equivalent factor graph and then using the max-product algorithm implemented in the libDAI C++ library [28] to solve it.

### Comparison of optimal amino acid sequences with native sequences

Most protein design methods, including the one described here, assume a fixed backbone structure because accounting for backbone flexibility would dramatically increase the number of degrees of freedom to be sampled. However, X-ray crystallographic studies have shown that proteins accommodate mutations in core residues by adjustments in the backbone structure [29]. This raises the question of how much bias the fixed backbone approximation introduces into protein design methods and whether this bias can be partially corrected by the backbone flexibility implicit in the smooth 6D residue pair scoring functions introduced here. This was studied by comparing the sequence identity between optimal sequences for backbone structures from two homologous proteins within the same family. A test set of protein structure pairs was compiled for this purpose by randomly selecting pairs of protein structures from the same HOMSTRAD [30] family. Each pair was selected from a different family and the final set contained a total of 407 protein pairs. HOMSTRAD families contain proteins with significant sequence or structure similarity so that each pair of proteins are presumably homologous.

We also studied the related question of how similar the optimal sequence is to the native sequence and how this similarity is affected by the protein design method. A previous study by Kuhlman and Baker [31] investigated this question using a fixed backbone design protocol that involved Monte Carlo sampling of both side chain conformations and residue types using all-atom energy based scoring with ROSETTA. The conclusion was that the sequence similarity between the optimal and native sequences is high, with identical residues for 51% of the buried core residues and 27% of all residues in the set of 108 backbone structures considered. However, the fixed backbone structure may lead to higher apparent sequence similarity because it is unable to accommodate as many mutations, primarily because of steric clashes in the protein core. In addition, many proteins share significant structure similarity without having any recognizable sequence similarity [32,33]. In these cases, the similarity between the optimal and native sequences must be low.

Both of these questions were examined by comparing the results using the protein design method described in the previous section, involving BP optimization using the 6D residue pair scores, with the results obtained using the ROSETTA program [18]. The ROSETTA optimal sequences were obtained using the default fixed backbone design algorithm implemented in Rosetta version 3.0. The two questions were investigated by comparing (1) the similarities between the optimal sequences for each pair of proteins in the same HOMSTRAD family (interstructure similarity) and (2) the similarities between the optimal and native sequences for a given backbone structure (intrastructure similarity), respectively. Sequence similarities were calculated both for all residues and for only core residues, which were defined as in Ref. [31] as those residues with > 20 C_*β *_atoms within 10 Å. Separate sequence similarities were also calculated separately for high-resolution (< 2.5 Å) and low-resolution (≥ 2.5 Å) protein structures. The results are shown in Table 5. First, both the intrastructure and interstructure similarities were higher for the core residues. This trend agrees with that observed in Ref. [31] and can be explained by the fewer allowed residue substitutions in the protein core due to stricter constraints resulting from van der Waals, hydrophobic, and to a lesser degree hydrogen bonding interactions [34]. Another clear trend that is apparent from these results is that the intrastructure similarity is higher for ROSETTA than the BP method. However, in contrast, the median interstructure similarity for BP derived optimal sequences are almost identical to that obtained by ROSETTA. If these latter results are further broken down by the resolution of the protein structures then it is seen that ROSETTA designed sequences have higher interstructure similarities for low-resolution structures whereas the BP method has higher interstructure similarities for high-resolution structures. One interpretation of these results is that the scoring functions have a smoother dependence on the backbone structure and thus yield more consistent optimal sequences for the similar backbones of homologous proteins. This interpretation is also supported by the lower dependence of the BP method on the resolution for both the intrastructure and interstructure similarities. Furthermore, the lower interstructure similarity for the BP method may be partly due to the fact that a fold is actually more permissive of different sequences once backbone flexibility is accounted for, rather than simply the expected lower accuracy of the residue pair scoring functions compared with the all-atom modeling of ROSETTA. In conclusion, even though both design methods rely on a fixed backbone approximation, the protein design method using the residue pair scoring functions yields optimal sequences that are more robust to the small variations in backbone structure that occur within protein families.

**Table 5:** Similarities between the optimal and native sequences (intrastructure) and similarities between the optimal sequences for a pair of proteins in the same HOMSTRAD family (interstructure)

	All residues			Core residues only		
	
	All structures	RMSD < 2.5 Å	RMSD ≥ 2.5 Å	All structures	RMSD < 2.5 Å	RMSD ≥ 2.5 Å
BP median %ID to	13.4%	13.5%	13.2%	17.6%	17.6%	17.8%
native						
ROSETTA median	25.9%	26.5%	23.6%	35.6%	36.2%	33.2%
%ID to native						
BP median	22.4%	24.0%	17.7%	29.4%	30.8%	25.3%
interstructure %ID						
ROSETTA median	22.8%	25.4%	17.2%	29.8%	33.6%	21.4%
interstructure %ID						
BP interstructure	183 (45%)	118 (41%)	65 (56%)	182 (45%)	116 (40%)	66 (57%)
%ID > ROSETTA						
interstructure %ID						
BP interstructure	222 (55%)	171 (59%)	51 (44%)	203 (50%)	161 (55%)	42 (36%)
%ID < ROSETTA						
interstructure %ID						
BP interstructure	2 (0.49%)	2 (0.70%)	0 (0%)	22 (5.4%)	14 (4.8%)	8 (6.9%)
%ID = ROSETTA						
interstructure %ID						

The similarities were calculated as percent sequence identity (%ID). The optimal sequences were calculated using the 6D residue pair scoring functions with Belief Propagation (BP) and using the ROSETTA program. The last three rows give the number of structure pairs for which the BP sequence similarity was less than, greater than, or equal to the similarity calculated using ROSETTA. The calculations were performed for a total of 407 structure pairs, each in a different HOMSTRAD protein family.

## Conclusions

The performance of 1D, 3D, and 6D residue pair scoring functions that depend only on the protein backbone structure were compared by their ability to identify correct threading solutions in a large benchmark set of proteins. The accuracy was found to improve as the dimension increased, with 6D scoring functions the most accurate. The orientation-dependent scoring functions were also found to achieve higher accuracy than several side chain-dependent empirical potentials on this benchmark set. This is remarkable since the backbone-only scoring functions do not require prediction of the side chain conformations, which is computationally demanding, particularly for many protein design applications. Interestingly, a previous study [35] also found that a backbone-only statistical potential outperformed a side chain-dependent potential, although the potential was derived and evaluated using decoy sets appropriate for folding rather than for fixed backbone design. We also found that averaging the threading scores for the query sequence and aligned segments of homologous sequences further improved the accuracy for protein threading applications. Incidentally, because those results were obtained for membrane proteins, they also demonstrated that the scoring functions can be successfully applied to the TM regions of membrane proteins even though they were derived from a data set containing predominantly non-membrane proteins. The applicability of the scoring functions to both membrane and non-membrane proteins is likely due to the fact that most of the residue pairs involve buried residues, which are not strongly affected by the distinct physiochemical environments of these two classes of proteins. However, direct comparison between the performance of the scoring functions on membrane and non-membrane proteins is needed to conclusively confirm this hypothesis. In addition, comparisons of predicted optimal amino acid sequences for pairs of similar backbone structures within the same protein families revealed that the 6D scoring functions were more robust to the small backbone rearrangements observed between homologous proteins than fixed-backbone all-atom modeling. Thus the 6D scoring functions should perform well in actual protein design applications in which the backbone structures of the designed and template proteins are expected to differ slightly.

Several possible extensions of this work are possible. First, as expected by the geometrical dependence of their physical interactions, accounting for the relative position and orientation of residue side chains should improve the accuracy of empirical scoring functions that depend on side chain conformations. Several previous studies have investigated such scoring functions, but with fewer relative degrees of freedom [9-11]. The same method used here for fitting 3D and 6D backbone-only scoring functions with GMMs could also be applied to such side chain conformation-dependent scoring functions. One difficulty is deciding the best choice of reference coordinate systems for larger flexible side chains. Second, the same methodology described here for deriving scoring functions for protein threading could be directly applied to derive similar residue pair scoring functions for other modeling tasks, such as *ab initio *protein structure prediction or protein-protein docking, by using the appropriate set of decoy structures. Finally, as mentioned earlier, the residue pair scoring functions can be employed in a fixed-backbone protein design procedure to generate initial protein sequences that are then subjected to all-atom optimization and scoring in order to select the optimal solutions.

## Methods

### Protein structures for training and evaluation

A set of Protein Data Bank (PDB) protein structures for which no two proteins share more than 25% sequence identity was obtained from the PDBSELECT database [36]. Protein structures with less than 50 residue contacts (defined below) were observed to be predominantly isolated alpha helices and so were removed from the set resulting in a total of 3328 structures. These structures of diverse proteins were then divided into ten approximately equal size sets for cross-validation.

### Residue pair relative position and orientation variables

Local coordinates at each residue are defined by axis unit vectors, , , and , which are centered at the respective C_*β *_atoms, as:(3)

so that the z-axis is along the C_*β*_-C_*α *_bond. A virtual C_*β *_atom was added to all glycine residues by assuming ideal tetrahedral bond central angles of  about C_*α *_and a typical C_*β*_-C_*α *_bond length of 1.53 Å. Six variables, {*r, θ, ϕ, α, β, γ*}, completely specify the relative position and orientation of the two residue backbones. The residue pair distance, r, is defined by the C_*β *_separation. The angles *θ *and *ϕ *are the polar angles of the residue #2 C_*β *_atom position in the residue #1 coordinate system. Finally, the relative orientation of the two coordinate systems fixed at each residue is described by three Euler angles, *α, β*, and *γ*. These Euler angles were calculated from the rotation matrix **M **between coordinate system #1 and coordinate system #2 with elements **M**_*ij *_= (**u**_2_)_*i*_·(**u**_1_)_*j*_, with , as(4)

in which the step function is defined by Θ (×) = 1 for ×>0 and 0 otherwise. Defining the vector connecting the origin of coordinate system #1 to the origin of coordinate system #2 as **v**_12 _then the remaining variables describing the relative position of residue #2 with respect to residue #1 are calculated as(5)

### Empirical scoring function as a Naïve Bayes classifier

The goal of the scoring function is to discriminate between two classes of protein structures: near-native (correct) structures and non-native (incorrect) structures. The input data for the query protein structure consists of the set of residue pair types and their relative orientation, *D *≡ {(*T*_*i*_, *T*_*j*_, **Ω**_*ij*_), *i *= 1, ..., *N*_residues_, *j *= *i *+ 1, ..., *N*_residues_}. The data for each residue pair is (*T*_*i*_, *T*_*j*_, **Ω**_*ij*_) with *T*_*i *_the amino acid type of residue number *i *and (*i, j*) are the residue numbers of neighboring pairs within a fixed cutoff separation of r_max _= 10 Å. The relative position and orientation of residue pair (*i, j*) are specified by either all 6 variables described above or a subset thereof:(6)

Using the Bayes theorem, the identity(7)

expresses the ratio of class posterior probabilities as the product of the class conditional probabilities of the data and the ratio of prior class probabilities. According to the maximum *a posteriori *(MAP) criterion, the protein structure is then classified as correct if the posterior ratio is greater than 1 or as incorrect if it is less than 1.

In a Naïve Bayes classifier, the class conditional probability of the data is approximated as a product of the probabilities for individual variables, *e.g*. the variables are considered independent, so that(8)

in which *S*_pair _(*T*_*i*_, *T*_*j*_, **Ω**_*ij*_) is defined as the log-odds residue pair score for residues *i *and *j*, which have types *T*_*i *_and *T*_*j *_and relative coordinates **Ω**_*ij*_. The prior probability ratio is defined as(9)

Classification by MAP then implies that the structure is classified as native if *S*_pair _>-log(*R*_prior_) and non-native if *S*_pair _<-log(*R*_prior_).

Log-odds scores are often converted into physical energy values using the Boltzmann distribution [37]. In spite of problems with their physical interpretation [38], such energy values may be useful when they are to be combined with physical energies from, for example, experimental data or force field calculations. However, one advantage of retaining log-odds scores is that the score cutoff has a useful interpretation in terms of Bayesian prior probabilities, as shown above. In any case, because the Boltzmann factor, exp(-*E*/*kT*), is monotonic, these two interpretations are operationally equivalent in terms of discriminating correct from incorrect solutions using a score cutoff.

The remaining task is to estimate the log-odds scores using training data. *S*_pair _(*T*_*i*_, *T*_*j*_, **Ω**_*ij*_) using training data. It should be noted that because the volume element depends on the relative angular coordinates for the 3D and 6D cases, a Jacobian factor is required. However, because the conditional probabilities in the numerator and denominator of Eq. (8) are evaluated at the same relative coordinates these factors cancel. The above relations are quite general and the choice of non-native data, and hence background probability distribution, will depend on the discrimination task. In this study, we are interested in identifying native, or stable, protein structures from among other decoy structures with the same backbone geometry but different residues. The resulting scores are then appropriate for both fold prediction by threading and protein design. The log-odds scores were calculated by estimating the class conditional probability distributions, *P *(*T*_*i*_, *T*_*j*_, **Ω**_*ij*_| native) and *P *(*T*_*i*_, *T*_*j*_, **Ω**_*ij*_| non-native), using two different density estimation methods applied to the native and non-native training data, respectively. The native distribution for a particular residue type pair was fit using the relative position and orientation of those residue types in native protein structures while the non-native distribution was fit to the relative position and orientation for all residue pairs in native backbone structures, regardless of their types. Thus the non-native distributions are actually independent of residue types and so equal to a common distribution, or *P *(*T*_*i*_, *T*_*j*_, **Ω**_*ij*_| non-native) = *P*_*nn *_(**Ω**_*ij*_). Kernel Density Estimation was used to fit the 1D scores and Gaussian Mixture Models were used to fit the 3D and 6D scores.

### Deriving 1D distributions using Kernel Density Estimation

Histograms of the residue pair separation distances have been commonly used to derive distance-dependent residue pair scores. Kernel Density Estimation (KDE) is a technique that has the advantages over histograms of yielding a smooth density estimate as well as a faster asymptotic rate of convergence of *O*(*n*^-4/5^) as compared with *O*(*n*^-2/3^) for histograms as the number of data points, *n*, is increased [39].

KDE fits the probability density function (pdf) *P*(*r*) as a sum of kernel functions centered about each data point(10)

In particular, Gaussian functions of uniform bandwidth, , were used in this study in order to estimate the distribution of residue separation distances. KDE was calculated in R [40] with the default bandwidth parameter *λ *calculated by the method of Ref. [41]. The resulting density was then approximated using linear interpolation of values calculated on a regular grid for computational efficiency.

### Deriving 3D and 6D distributions using Gaussian Mixture Models for density estimation

The "curse of dimensionality" [42] makes estimating the probability densities in the full 3- and 6-dimensional relative coordinate spaces challenging. Histograms are infeasible because the number of bins increases exponentially with the number of dimensions, assuming equal length in each bin dimension, and so the quantity of training data is inadequate for 3- or 6-dimensional fitting. Kernel density estimation is also challenging for two reasons. First, the computational resources, both CPU time and memory, are prohibitive for a straightforward implementation. Second, the standard methods for estimating the Gaussian kernel bandwidth matrix, which contains *D*(*D *+ 1)/2 parameters in the general D-dimensional case, are not feasible in higher dimensions. Instead we have chosen to use Gaussian mixture models (GMMs) because they can accurately and efficiently fit the high-dimensional densities required for the residue pair scores.

A Gaussian mixture model with *N*_comp _components estimates the pdf as(11)

in which(12)

is a multidimensional Gaussian function and the mixture proportions satisfy . The model parameters are usually estimated by maximum likelihood using the Expectation-Maximization (EM) algorithm [43,44].

A general problem with statistical models is choosing the optimal complexity of the model, *i.e*. number of parameters in the model. The accuracy of the model, as reflected in the GMM log-likelihood, steadily improves as the number of parameters is increased, even though the accuracy on novel data, not used for fitting the model, is optimal at a finite number of parameters. In order to fit the 3- and 6-dimensional densities, Gaussian kernels with full-rank covariance matrices Σ_*k *_were used and the optimal number of components was chosen by minimizing the Bayes Information Criterion (BIC) [14]

As the number of model parameters is increased, the first term in the BIC decreases while the second term increases so that a minimum is achieved at an intermediate value. The R package MCLUST [45,46] was used to search for the best GMMs with up to 100 components. In all except a few cases the optimal number of components was less than the maximum.

### Single residue scoring function

In addition to the pairwise residue scoring function described above, a single residue scoring function was included in the BP prediction of an optimal amino acid sequence (see Eq. (2)). The single residue score depends on two residue properties, solvent accessibility and secondary structure, and, like the pairwise case, is a log-odds score. Each residue was assigned to two solvent accessibility classes, buried or surface, depending on whether its relative solvent accessible surface area (SASA) is < 0.01 or ≥ 0.01, respectively. The relative SASA was calculated as ratio of the SASA to the maximum SASA as defined in [47]. The secondary structure was classified as an *α*-helix (H), *β*-strand (E), or other (C). The score was then calculated as(13)

in which the solvent accessibility class of residue i is *Acc*_*i *_∊{ buried, surface} and the secondary structure of residue i is *SS*_*i *_∊{ H, E, C}. The probabilities *P*(*Acc*_*i*_, *SS*_*i *_| native) and *P*(*Acc*_*i*_, *SS*_*i *_| non-native) were estimated from the benchmark set of protein structures with the native sequences and shuffled sequences, respectively. The single residue properties are assumed to be independent of the pairwise geometrical properties so that, when both scores are used, the total score is their sum.

### Non-redundant set of contacting transmembrane helix structures

A set of high-resolution structures of interacting transmembrane (TM) helix pairs were extracted from PDB membrane protein structures in order to test whether including homologs improves threading accuracy, as described in the Results section. TM segment boundaries were determined by the PDBTM database [48,49]. The included TM helix pairs were non-redundant both with respect to their amino acid sequences, by selecting sequences with < 30% sequence identity, and structure, by clustering them by structural similarity and choosing a single representative structure from each cluster. Clustering was performed by calculating all pairwise similarities between different helix pairs, as measured by the C_*α *_RMSDs of their aligned segments, and then clustering them based on these RMSD values. The aligned segments were required to be at least 10 residues long and contain all contacting residues. All possible segments in corresponding helices that satisfied these criteria were superimposed using the Kabsch algorithm [50]. Also both possible correspondences of helices in each helix pair were tried, *i.e*. (A→ A', B→ B') and (A→ B', B→ A') for helix pairs (A, B) and (A', B'). Once the similarity matrix was calculated, the helix pairs were then clustered using the robust Partioning Around Medoids (PAM) algorithm [51]. In addition to the cluster assignments, the PAM algorithm outputs a medoid for each cluster. A medoid is the member of each cluster that has the minimum average dissimilarity to all other cluster members. Thus, in a sense, it can be considered the central object in each cluster. Because of this desirable property, the medoid helix pairs in each cluster were chosen as the representative structures that comprised the set. The number of clusters, 78, was chosen so that the maximum RMSD between a medoid and any other helix pair in the same cluster was 2.5 Å.

### Homologous sequences for TM helices

Aligned sequences of homologs were then collected for each TM helix in the non-redundant set of 78 helix pairs. First BLAST [52] was used to search the NCBI nr protein sequence database using full-length protein sequences containing the helices (E-value cutoff = 10^-2^). Highly similar sequences were then removed using CD-HIT [53] with a 90% sequence identity cutoff and the remaining sequences aligned with MUSCLE [54]. Only gapless segments aligned to the TM helices were included in the final set of homologous sequences used for threading.

### Membrane depth-dependent scoring function

A previous study [55] derived a log-odds depth-dependent empirical potential for inserting different amino acid side chains into the membrane. The potential had a good correlation with experimental transfer free energy values and was able to reproduce the correct tilt angle of TM *α*-helices with respect to the membrane. We derived a similar log-odds scoring function from the propensities of different residue types in *α*-helical integral membrane proteins to occur at different depths in the membrane. The scoring function was added to the 6D residue pair scoring function for the prediction of TM *α*-helix associations described in the Results section.

First all *α*-helical TM segments were downloaded from the PDBTM database and a non-redundant set extracted using the CD-HIT program [53] with a 30% sequence identity cutoff. Residue depths were defined by the absolute distance, |z|, from the PDBTM predicted central membrane plane. The log-odds depth-dependent score for each amino acid type *i*, S_i_(z), was then calculated as(14)

in which P(z|aa_i_) is the pdf of amino acid type *i *appearing at membrane depth *z *and P(z) is the pdf of *any *residue appearing at depth *z*. Both pdfs are defined to be symmetric, *i.e*. P(-z|aa_i_) = P(z|aa_i_) and P(-z) = P(z). Unlike the previous study, which fit fixed functional forms to histograms of amino propensities as a function of depth, KDE was used to fit the pdfs, just as for the 1D (distance-dependent) residue pair scoring functions.

## Authors' contributions

AJB designed and performed the study, analyzed the results, and wrote the manuscript.
